# Supplementary material for: Subtype and cell type specific expression of lncRNAs provide insight into breast cancer
Source: Commun Biol. 2022 Aug 18;5:834. doi: 10.1038/s42003-022-03559-7 (PMC9388662; doi:10.1038/s42003-022-03559-7)
Supplement: Supplementary file 1 — Description of Additional Supplementary Files [file 42003_2022_3559_MOESM1_ESM.pdf]

Description of Additional Supplementary Files

File name: Supplementary Data 1
Description: Underlying data Fig. 1 and Supp.Fig 3.

File name: Supplementary Data 2
Description: Survival analysis.

File name: Supplementary Data 3
Description: Underlying data Fig. 2a-d.

File name: Supplementary Data 4
Description: Underlying data Fig. 2e-g.

File name: Supplementary Data 5
Description: lncRNAs associated to breast cancer cell lines.

File name: Supplementary Data 6
Description: Underlying data Fig. 4b-g.

File name: Supplementary Data 7
Description: Underlying data Fig. 5.

File name: Supplementary Data 8
Description: Sample IDs used in analysis.

File name: Supplementary Data 9
Description: SCAN-B kallisto TPM values.

File name: Supplementary Data 10
Description: TCGA-BRCA kallisto TPM values.
