# Supplementary material for: Subtype and cell type specific expression of lncRNAs provide insight into breast cancer
Source: Commun Biol. 2022 Aug 18;5:834. doi: 10.1038/s42003-022-03559-7 (PMC9388662; doi:10.1038/s42003-022-03559-7)
Supplement: Supplementary file 2 — Supplementary Information [file 42003_2022_3559_MOESM2_ESM.pdf]

Subtype and cell type specific expression of lncRNAs provide insight into breast cancer

Sunniva Bjørklund[1,2], Miriam Ragle Aure[1,2], Jari Häkkinen[3], Johan Vallon-Christersson[3], Surendra Kumar[4], Katrine Bull Evensen[2], Thomas Fleischer[2], Jörg Tost[5], Anthony Mathelier[1,6], Gyan Bhanot[7,8], Shridar Ganesan[8], Xavier Tekpli[1,2,*], Vessela N. Kristensen[1,9,*]

1) Department of Medical Genetics, Oslo University Hospital, Oslo, Norway
2) Department of Cancer Genetics, Institute for Cancer Research, Oslo University Hospital Radiumhospitalet, Oslo, Norway
3) Division of Oncology, Department of Clinical Sciences Lund, Lund University, Medicon Village, SE 22381, Lund, Sweden.
4) Department of Ocean Sciences, Memorial University of Newfoundland, St. John's. NL, Canada
5) Laboratory for Epigenetics and Environment, Centre National de Recherche en Génomique Humaine, CEA – Institut de Biologie Francois Jacob, University Paris Saclay, Evry, France
6) Centre for Molecular Medicine Norway (NCMM), Nordic EMBL Partnership, University of Oslo, 0349 Oslo, Norway
7) Department of Physics and Astronomy, Rutgers University, Piscataway, NJ 08854, USA.
8) Rutgers Cancer Institute of New Jersey, New Brunswick, NJ 08903, USA.
9) Institute of Clinical Medicine, University of Oslo, Oslo, Norway

[*] Jointly supervised the work, shared corresponding authors

Corresponding authors:
Prof. Vessela N. Kristensen
Email: v.n.kristensenedisin.uio.no
Dr. Xavier Tekpli
Email: xavier.tekpli@medisin.uio.no
Department of Medical Genetics
Oslo University Hospital
Oslo, Norway
Phone: +47 22 78 13 75

Supplementary Figures 1-15

Supplementary Figure 1

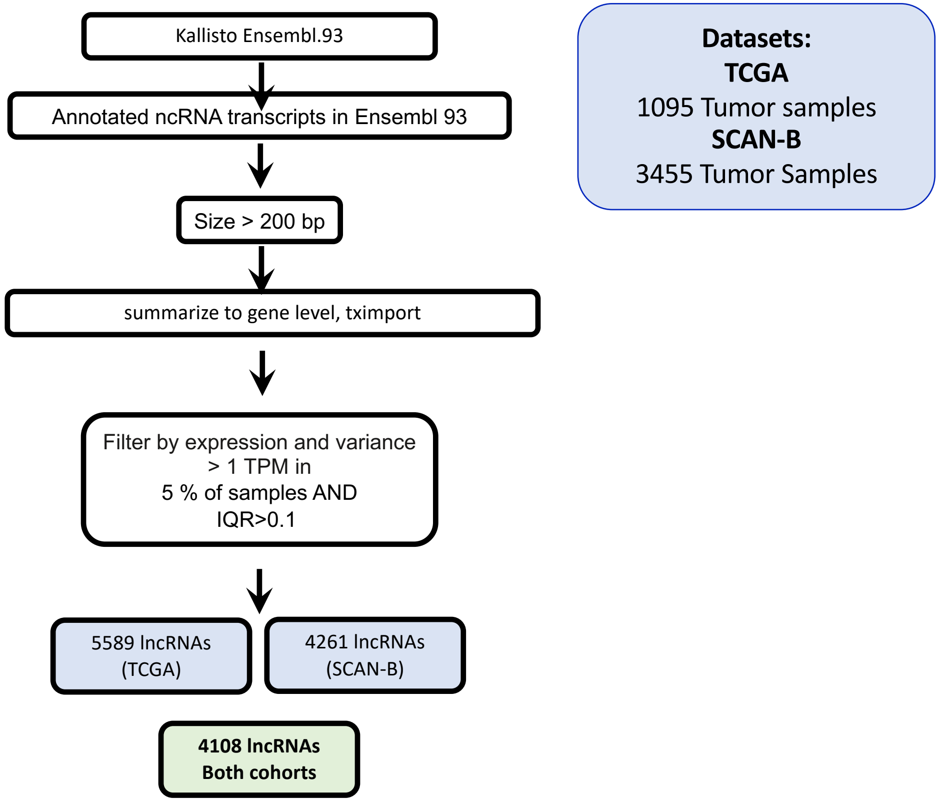

Supplementary Figure 1. lncRNA quantification.

All mRNAs and non-coding transcripts were quantified using Kallisto. Transcript level quantification was summarized to gene level using tximport. All lncRNAs with expression above 1 Transcripts Per Kilobase Million, found in more than 5% of samples in each cohort, and with a variance IQR >0.1 were further analyzed. In total 4108 lncRNAs were included in the downstream analysis.

Supplementary Figure 2

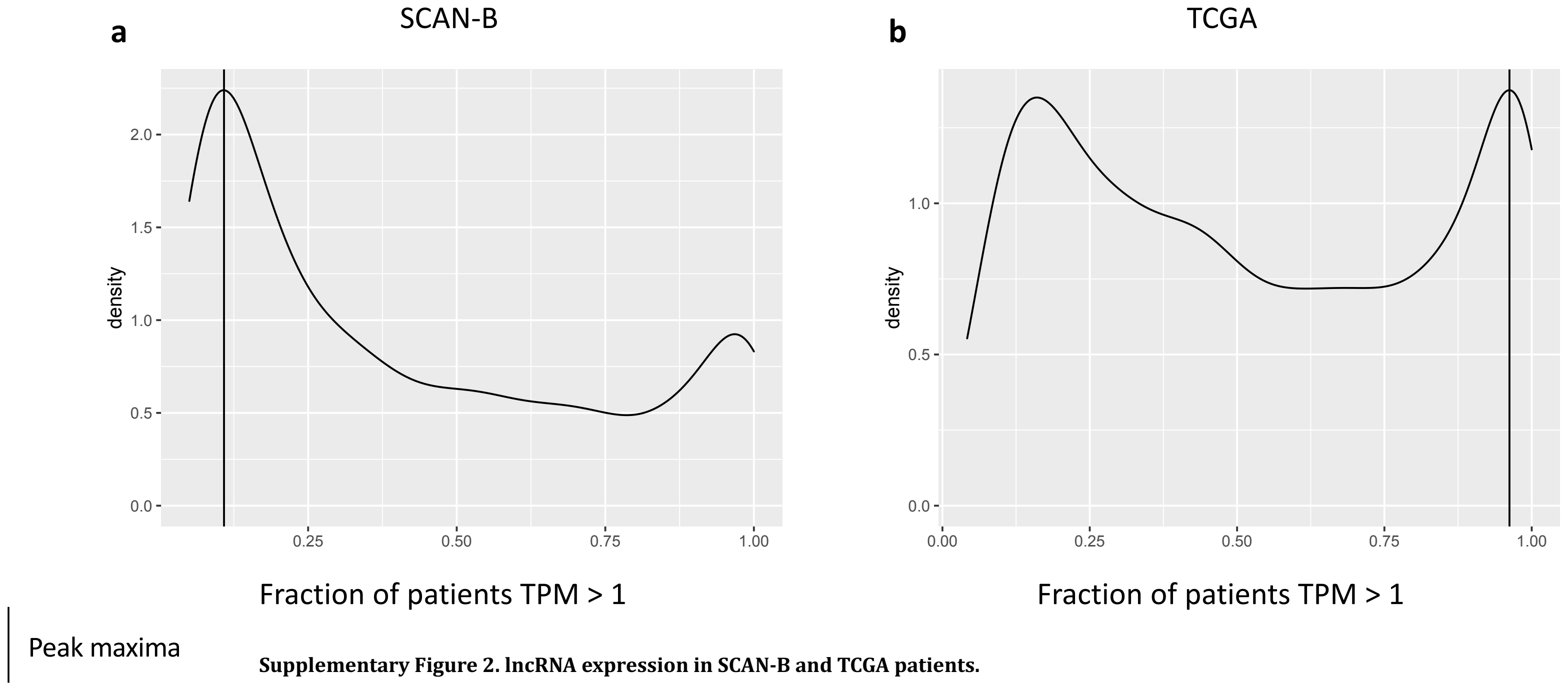

Supplementary Figure 2. lncRNA expression in SCAN-B and TCGA patients.

Density plot of the fractions of patients in **(a)** SCAN-B and **(b)** TCGA that express each of the 4108 lncRNAs at TPM > 1. Vertical lines represent the peak maxima using the which.max function in R .

Supplementary Figure 3

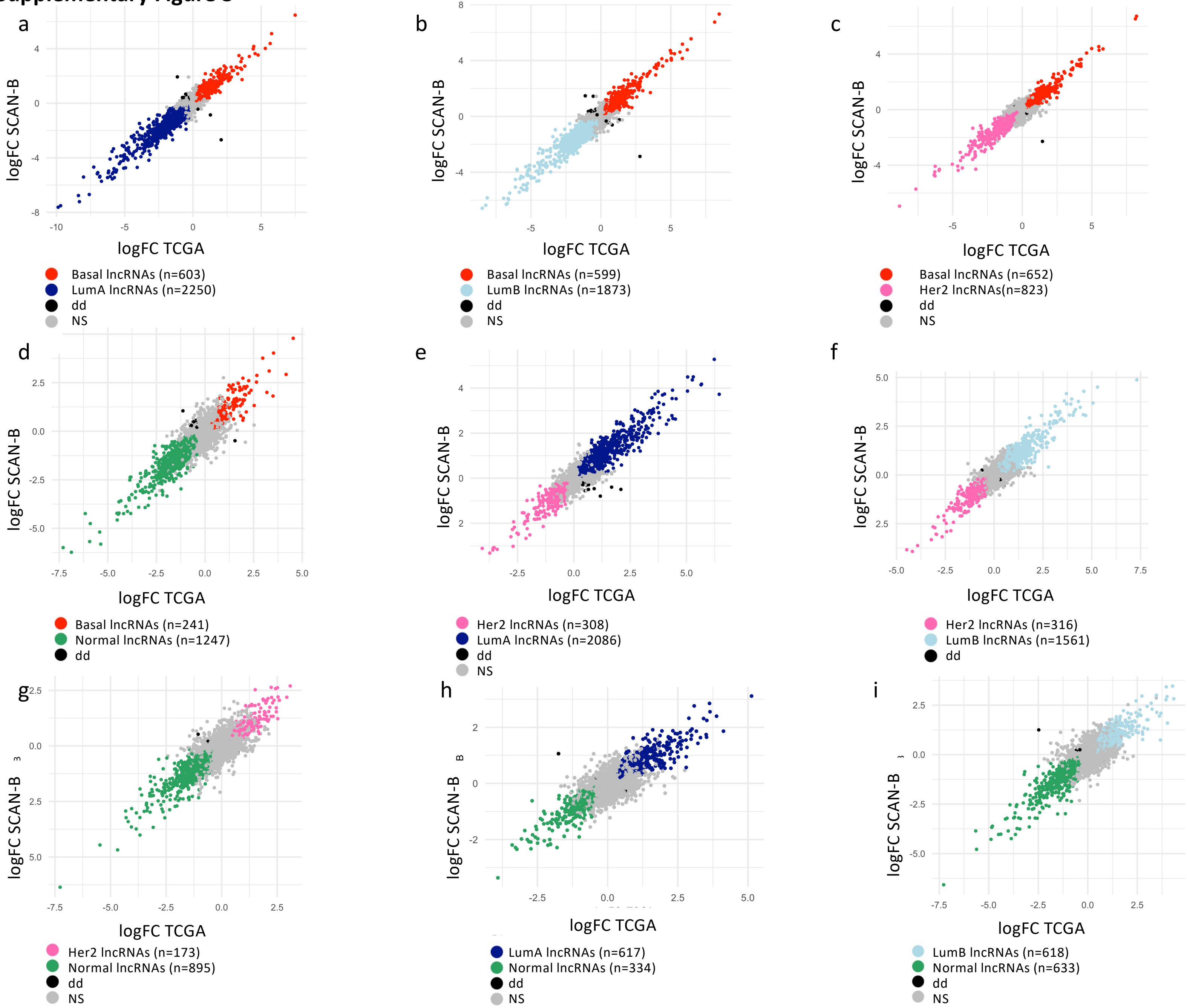

Supplementary Figure 3. Pairwise differential expression among breast cancer subtypes.
Log Fold Change between two groups from SCAN-B (x-axis) and TCGA (y-axis) calculated using a fitted Limma model (lmfit) and moderated t-statistic (eBayes). Coloured dots depict differentially expressed lncRNA (Benjamini-Hochberg adjusted p-value < 0.05) in both cohorts; gray dots shows lncRNAs with non-significant differential expression in one or both cohorts; black dots indicate lncRNAs with significant p-value but with different direction (dd, logFC sign) in the two cohorts.
(a) Basal-like *vs.* Luminal A, **(b)** Basal-like *vs.* Luminal B, **(c)** Basal-like *vs.* Her2-like, **(d)** Basal-like *vs.* Normal-like, **(e)** Luminal A *vs.* Her2-like, **(f)** Luminal B *vs.* Her2-like, **(g)** Her2-like *vs.* Normal-like, **(h)** Luminal A *vs.* Normal-like, **(i)** Luminal B *vs.* Normal-like.

Supplementary Figure 4

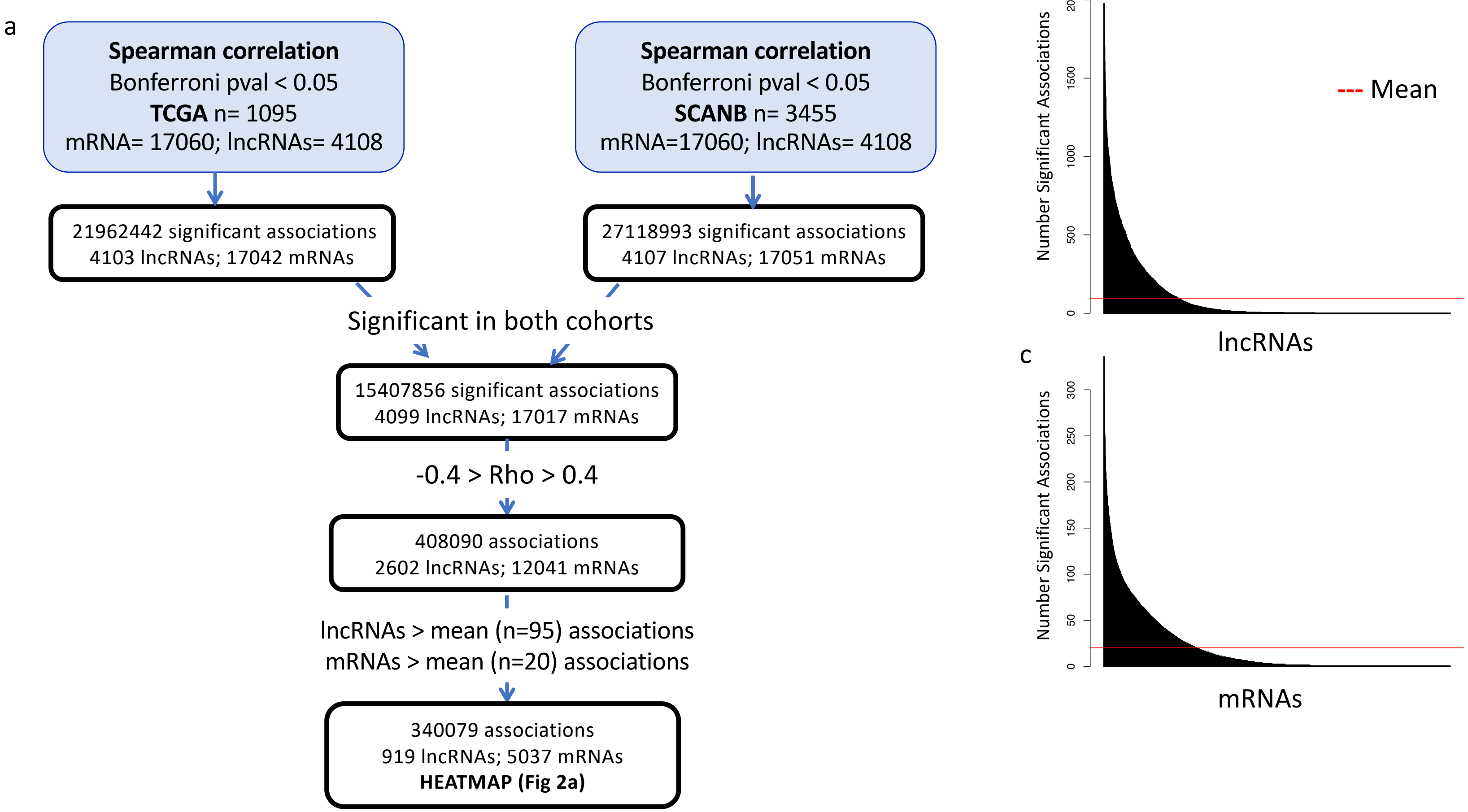

Supplementary Figure 4. lncRNA-mRNA correlation analysis.

(a) Outline of the correlation analysis between lncRNAs (n=4108) and mRNAs (n=17060). lncRNA-mRNA correlations with Bonferroni corrected p-value < 0.05 in both datasets are shown in Supplementary Data 3. To further cluster lncRNA-mRNA correlations (Figure 2a) only lncRNA-mRNA pairs with i) - 0.4 > Spearman rho > 0.4 in both datasets, and ii) lncRNA and mRNA associations with number of significant associations above average. On average each lncRNA was significantly correlated to the expression of 95 mRNAs (**b**), while each mRNA was on average correlated to 20 lncRNAs **(c)**.

Supplementary Figure 5

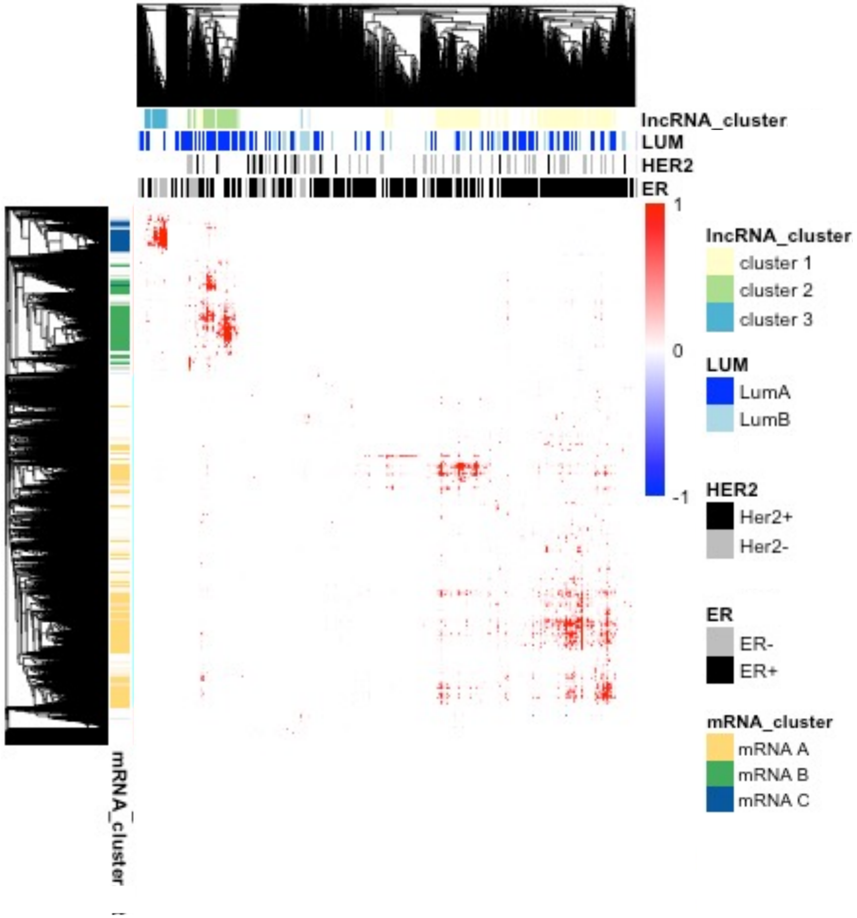

Supplementary Figure 5. Clustering of lncRNA into relevant pathways for breast cancer.

Hierarchical clustering of lncRNA-mRNA Spearman Correlation values (positive correlation in red, negative correlation in blue) following co-expression analysis between lncRNAs (n=4108) and protein coding mRNAs (n=17060). All lncRNA and mRNA with significant correlation (Bonferonni p-value < 0.05) in the TCGA and SCAN-B cohorts are used in the unsupervised clustering. Clusters from the main Figure 2a are included as row (mRNA_cluster) and column annotations (lncRNA_cluster). The scale (blue-red) represents Spearman correlation values that were binarized to -1/1 for negative and positive correlation respectively.

Supplementary Figure 6

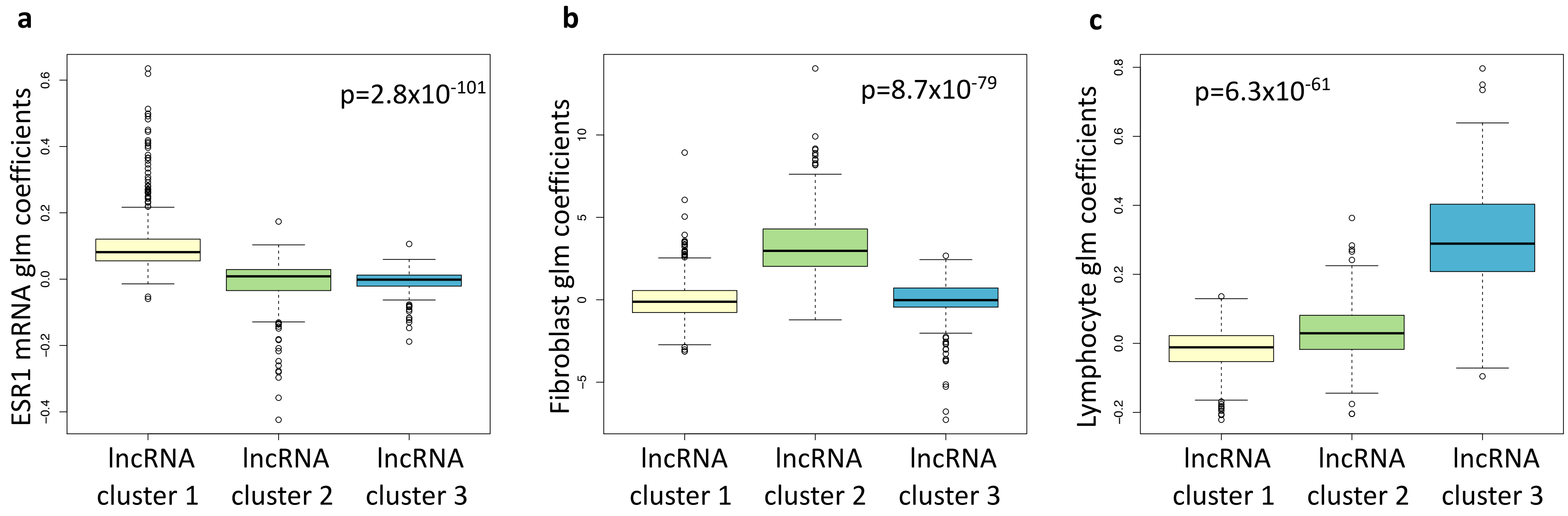

Supplementary Figure 6. Boxplot for the coefficient of the generalized linear model of the expression of lncRNA

Boxplot of the coefficients from the generalized linear model of the expression of lncRNA in the TCGA cohort using three variables into the model, *ESR1* mRNA (to reflect estrogen signaling **(a)**), fibroblast score (to infer fibroblast tumor content **(b)**) and lymphocyte score (to infer lymphocyte infiltration **(c)**). Each dot represents the coefficient for a variable and a lncRNA. Kruskal-Wallis test p-values denoted. The line within each box represents the median. Upper and lower edges of each box represent 75th and 25th percentile, respectively. The whiskers represent the lowest datum still within [1.5 × (75th – 25th percentile)] of the lower quartile, and the highest datum still within [1.5 × (75th – 25th percentile)] of the upper quartile.

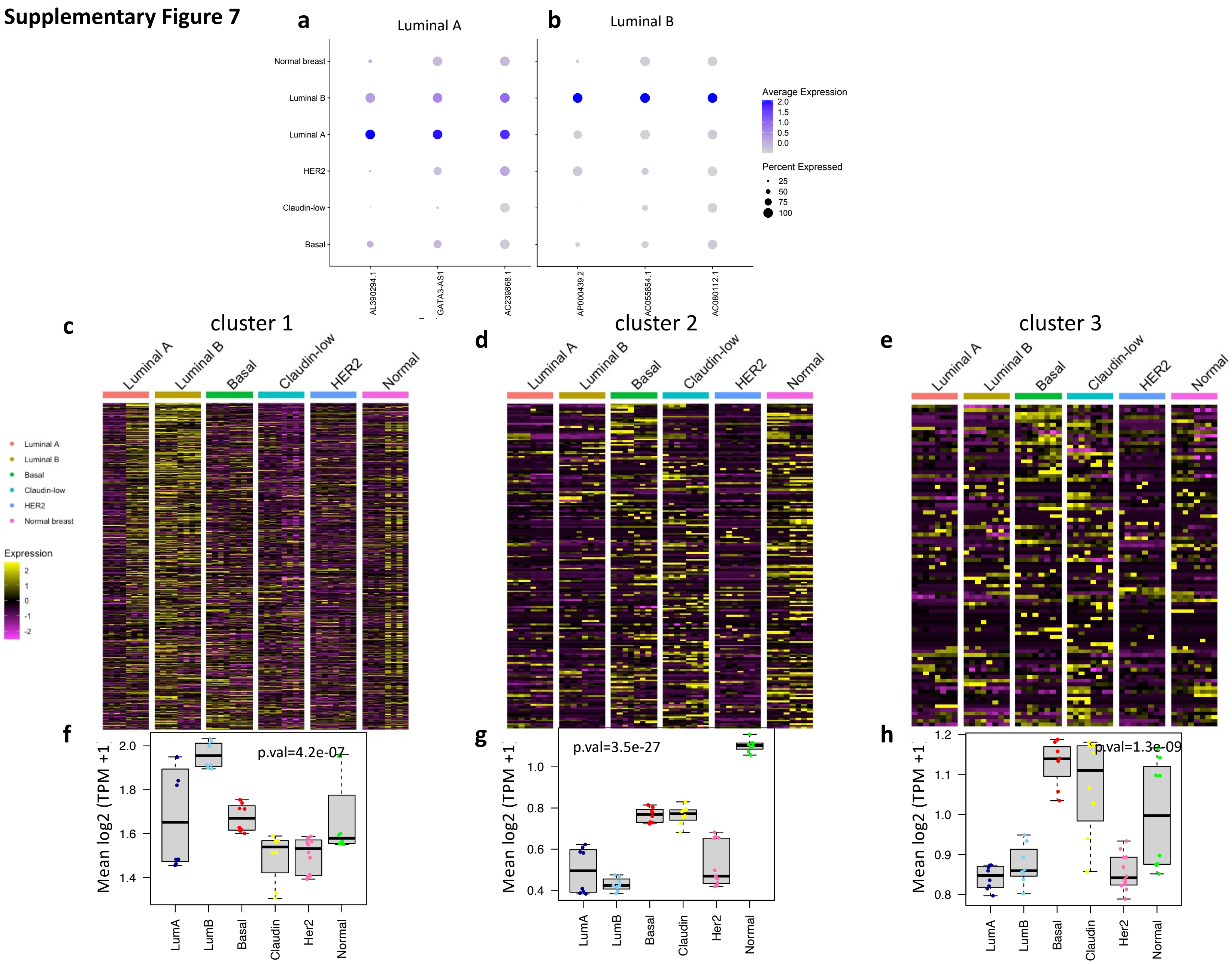

Supplementary Figure 7. lncRNA expression in Breast cancer cell lines.

Dot plots showing the top 3 differentially expressed lncRNAs in Luminal A (n=8) **(a)** and Luminal B (n=8) cell lines **(b)**. Size of the dot represents the percentage of samples expressing the lncRNA, while the colour of the dot reflects the average expression in each of the cell lines stratified by subtype. Each subtype is represented by two unique cell lines in replicates of 4 (dot).

Heatmaps showing expression of all lncRNAs in cluster 1 **(c)**, cluster 2 **(d)**, and cluster 3 **(e)** in breast cancer cell lines stratified by subtype. Each subtype is represented by n=8 replicates. The expression gradient (pink to yellow) represents scaled and centered log2 (TPM+1) values. Boxplot of the mean expression of all lncRNAs in cluster 1 **(f)**, cluster 2 **(g)**, and cluster 3 **(h)** in breast cancer cell lines of different subtypes. ANOVA test p values are shown. The line within each box represents the median. Upper and lower edges of each box represent 75th and 25th percentile, respectively. The whiskers represent the lowest datum still within [1.5 × (75th – 25th percentile)] of the lower quartile, and the highest datum still within [1.5 × (75th – 25th percentile)] of the upper quartile.

Supplementary Figure 8

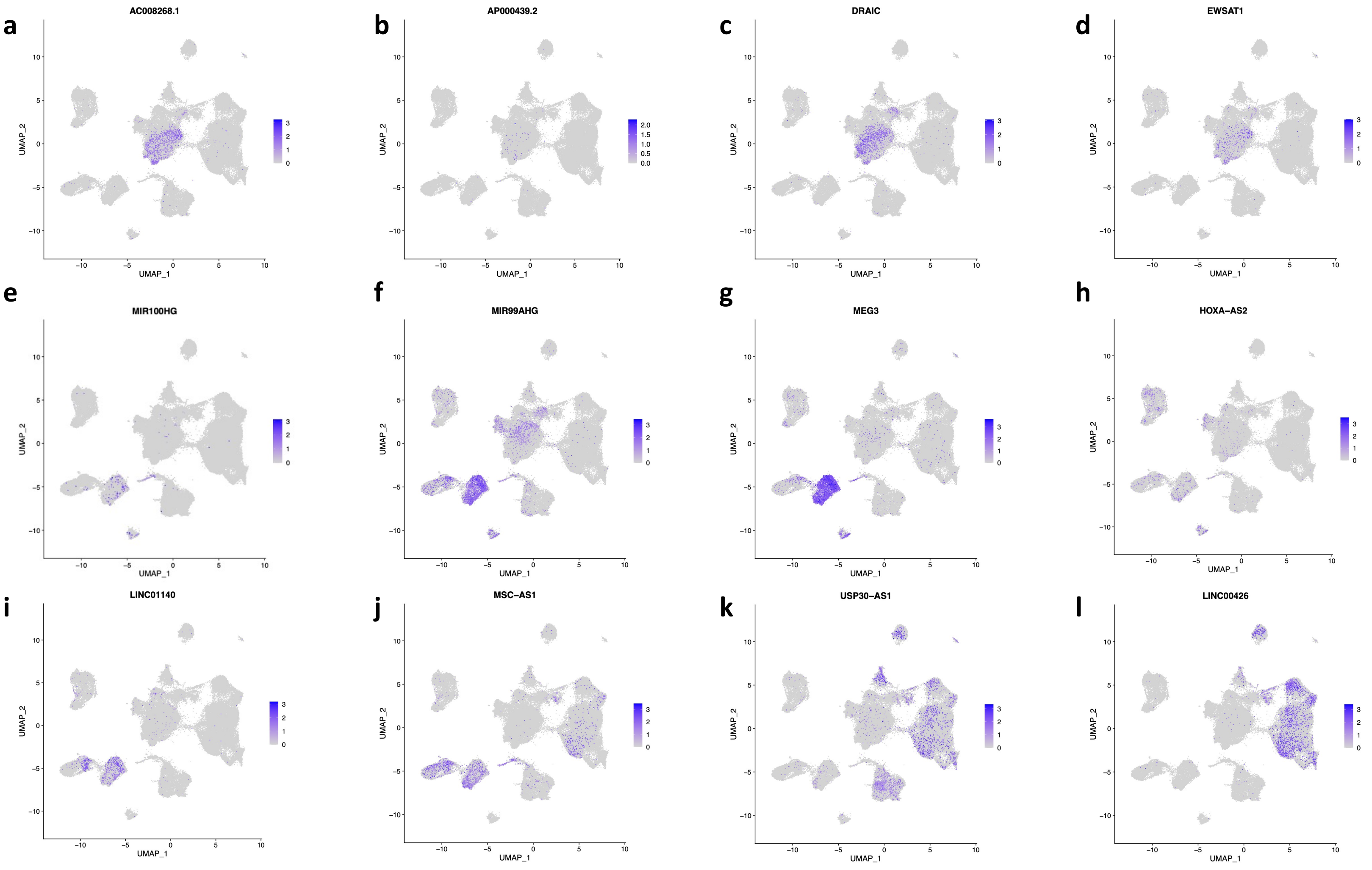

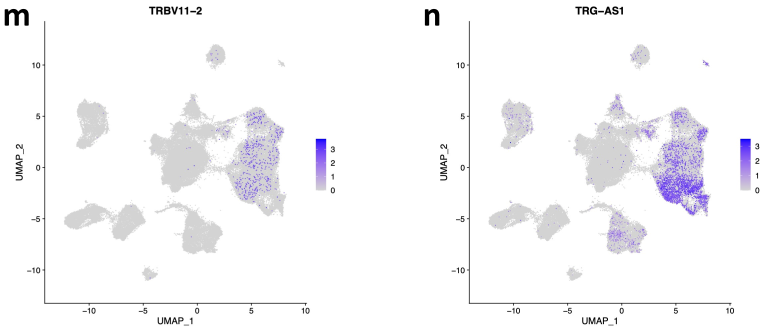

Supplementary Figure 8. lncRNA expression in single cell RNA-seq data

(a-n) Expression of high ranking lncRNAs from each lncRNA cluster (Figure 2a) plotted on the scRNA-seq UMAP. Cluster 1-lncRNAs **(a-d)**, cluster 2-lncRNAs **(e-j)**, and cluster 3-lncRNAs **(k-n)**. Colour gradient (purple) represents log normalized counts using scale.factor = 10000.

Supplementary Figure 9

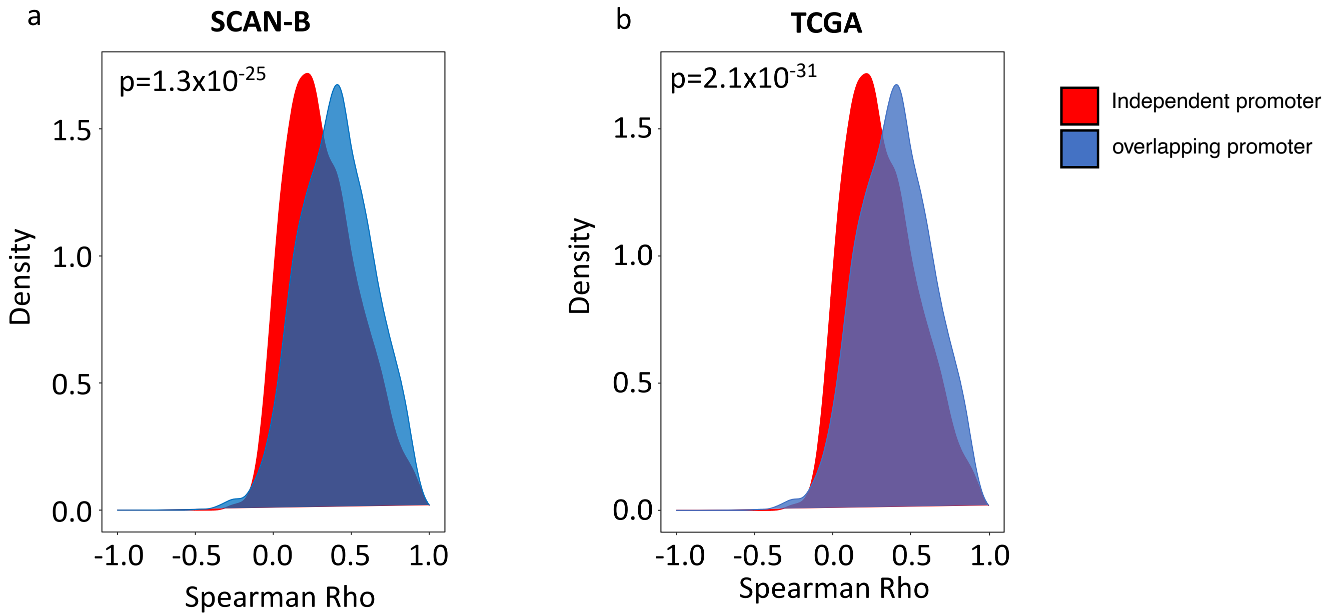

Supplementary Figure 9. lncRNA promoter annotation.

Density plot of the Spearman correlation rho value from **(a)** SCAN-B and **(b)** TCGA, from the expression of lncRNAs and its overlapping protein coding mRNA (overlapping lncRNA-promoter, blue). For non-overlapping promoter lncRNA with protein coding gene, the density plot of the Spearman rho with the nearest protein coding mRNA is plotted (independent lncRNA;pink). Pairwise t-test p-values denoted.

Supplementary Figure 10

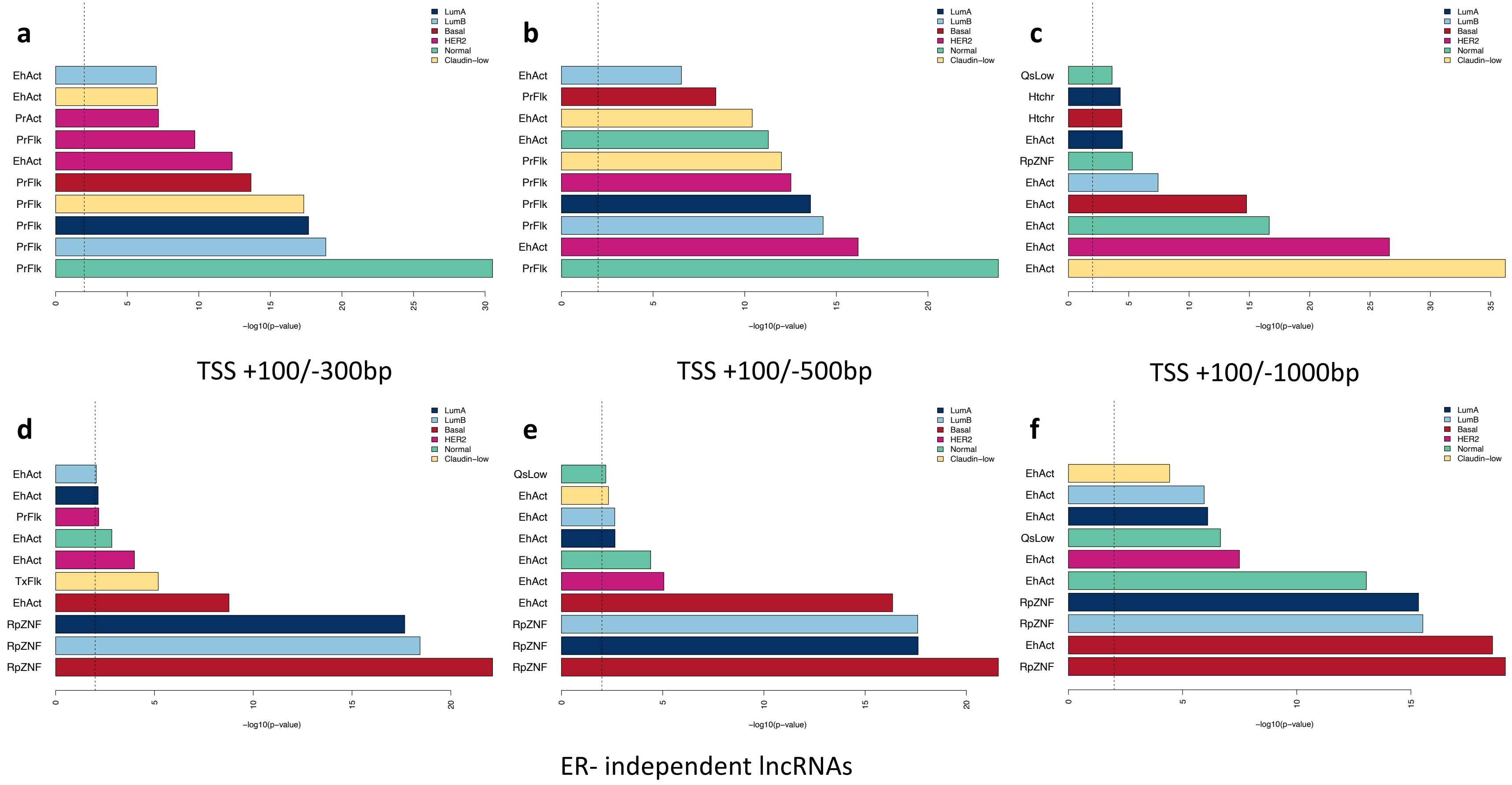

Supplementary Figure 10. ChromHMM annotation of lncRNA promoters.

Enrichment of lncRNA promoters across ChromHMM genome segmentation from breast cancer cell lines. Enrichment is calculated as the ratio between the frequency of lncRNA promoters found within a specific segment type, over the frequency of all lncRNA promoters within the same segment type. The length of the bars (x-axis) shows the log transformed BH corrected p-value from the hypergeometric test **(a-c)** promoters overexpressed in ER positive cases and **(d-f)** promoters overexpressed in ER negative cases. Enrichment is shown for expanding window sizes upstream of the TSS, -300bp **(a&d)**, -500bp **(b&e)**, and -1000bp **(c&f)**. Active Enhancer=EhAct, Active Promoter=PrAct, Repeat Zink Finger= RpZNF, Flanking Promoter region= PrFlk.

Supplementary Figure 11

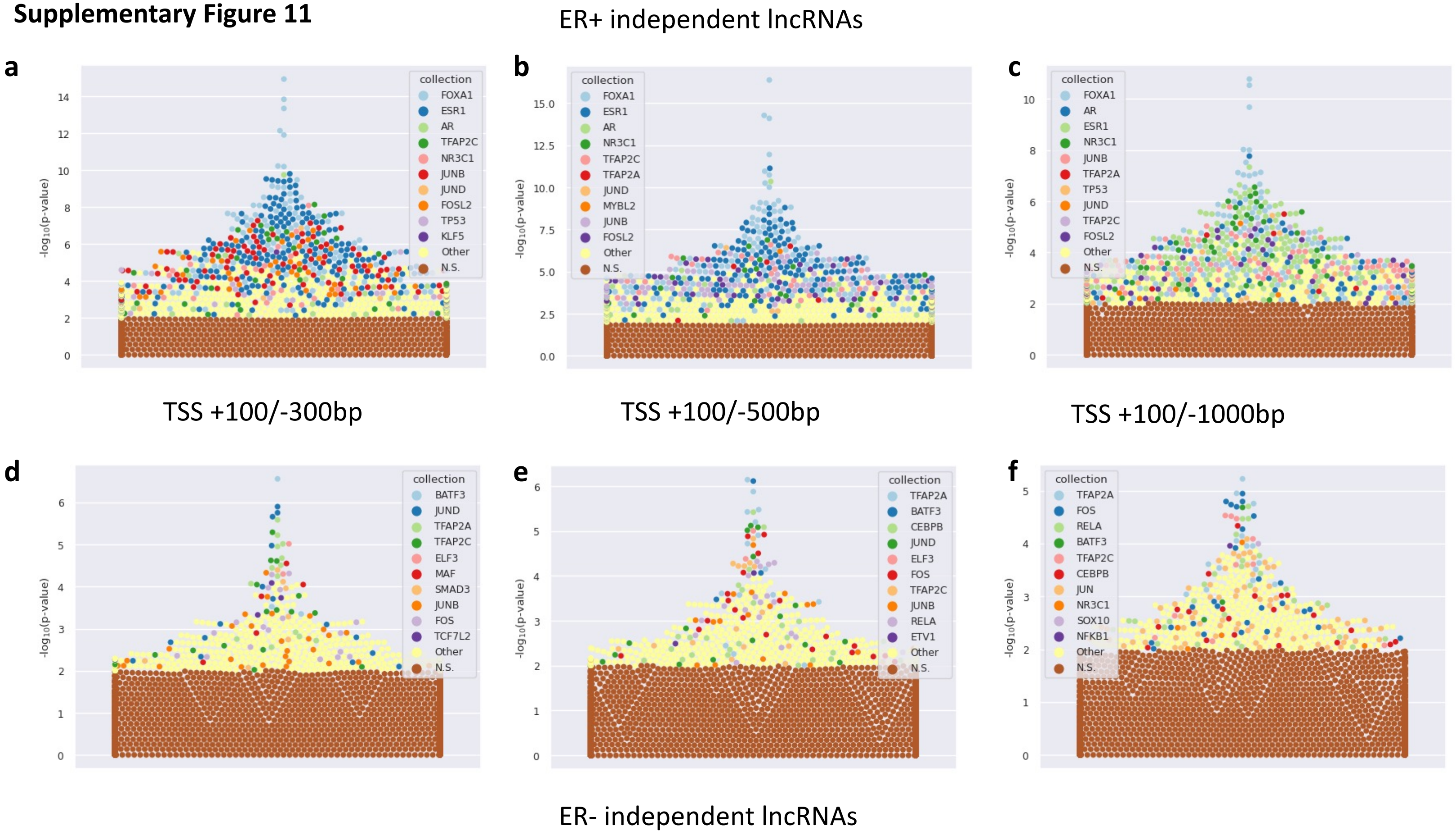

Supplementary Figure 11. TF binding at lncRNA promoters.

Swarm plot showing enrichment of TF binding sites (–(log10(p-value) using Fisher's exact tests) on the y-axis for specific sets of promoters according to UniBind. TF names of the top 10 enriched TF binding sites data sets are annotated by colours **(a-c)** promoters overexpressed in ER positive cases and **(d-f)** promoters overexpressed in ER negative cases. Enrichment is shown for expanding window sizes upstream of the TSS, -300bp **(a&d)**, -500bp **(b&e)**, and -1000bp **(c&f)**.

Supplementary Figure 12

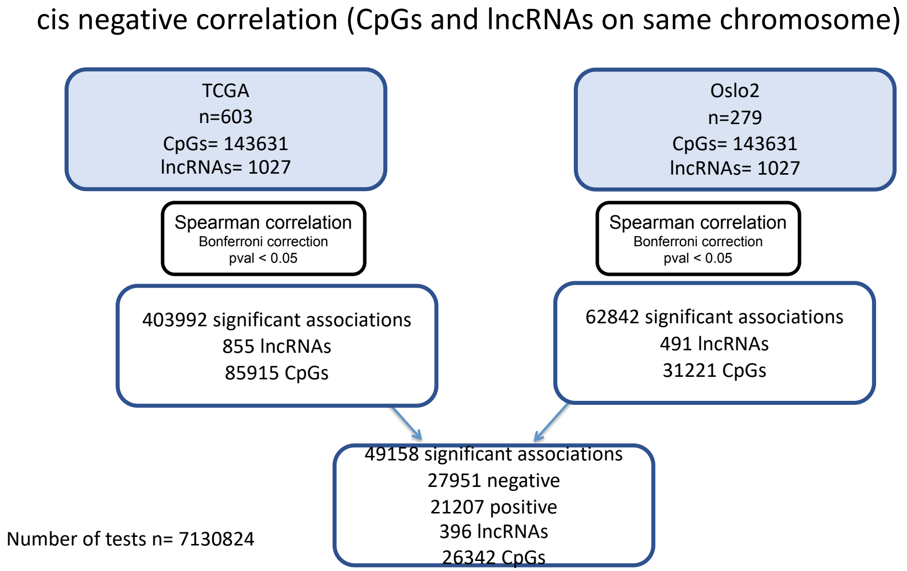

Supplementary Figure 12. Correlation analysis between levels of DNA methylation and lncRNA expression.

For CpGs and lncRNA on the same chromosome correlation between DNA methylation and lncRNA expression was performed for 1027 lncRNA found in both TCGA (n=603) and OLS2 (n=279), two cohorts for which both DNA methylation and lncRNA expression are available.

CpGs were filtered to have IQR>0.1 in both datasets. CpG-lncRNA associations with Bonferroni corrected p-values < 0.05 in both datasets were included in the downstream analysis.

Supplementary Figure 13

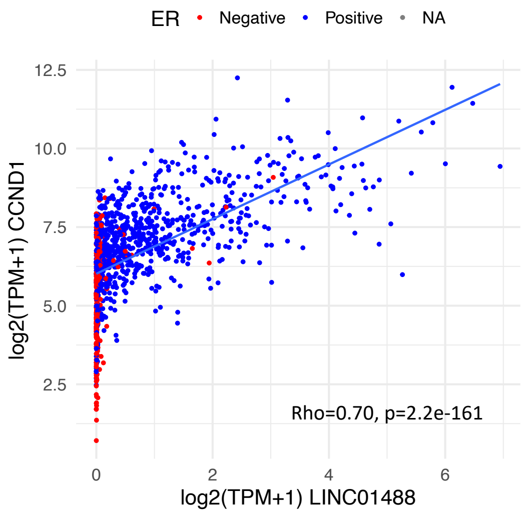

Supplementary Figure 13. lncRNA-mRNA correlation
Correlation plot of log2(TPM+1) *LINC01488* expression (x-axis) and log2(TPM+1) *CCND1* expression (y-axis) in ER positive (n=807) and ER negative (n=237) patients in the SCAN-B cohort. Rho and p-value from Spearman correlation is indicated.

Supplementary Figure 14

GATA3-AS1

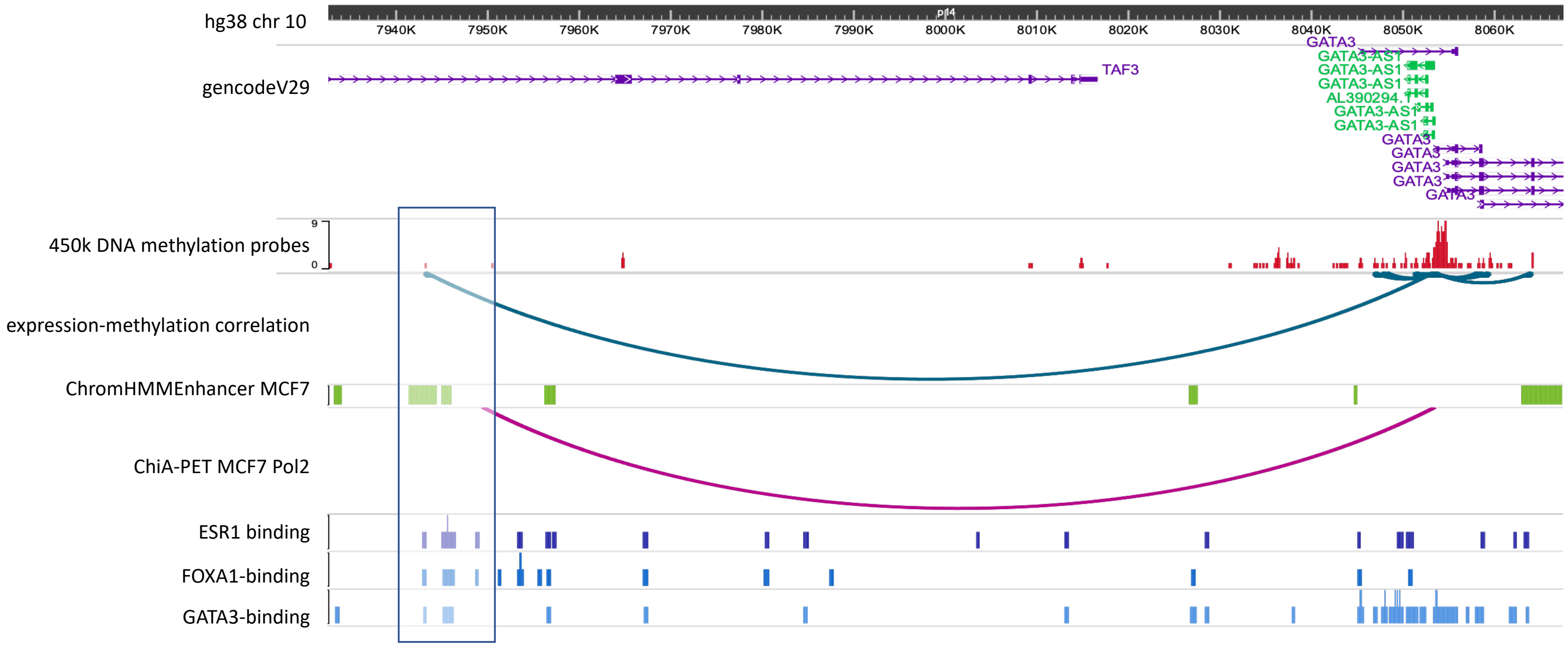

Supplementary Figure 14. Distal regulatory element in lncRNA loci

Graphical illustration of the *GATA3-AS1* locus annotated for different epigenomic tracks. CpGs measured by the 450k Illumina array are shown together with the significant negative correlations between levels of DNA methylation and lncRNA expression in the OSL2 and TCGA cohorts (blue arcs, expression-methylation correlation). ChromHMM Enhancer regions (active and genic) in the Mcf7 cell line (green), ChiA-PET polII loop connecting the TSS of the respective lncRNA to the CpG in the enhancer region. TF binding of ESR1 (dark blue), GATA3 (light blue), and FOXA1 (grey blue) from ChIP-seq experiments (ReMap).

Supplementary Figure 15

MIR29BCHG

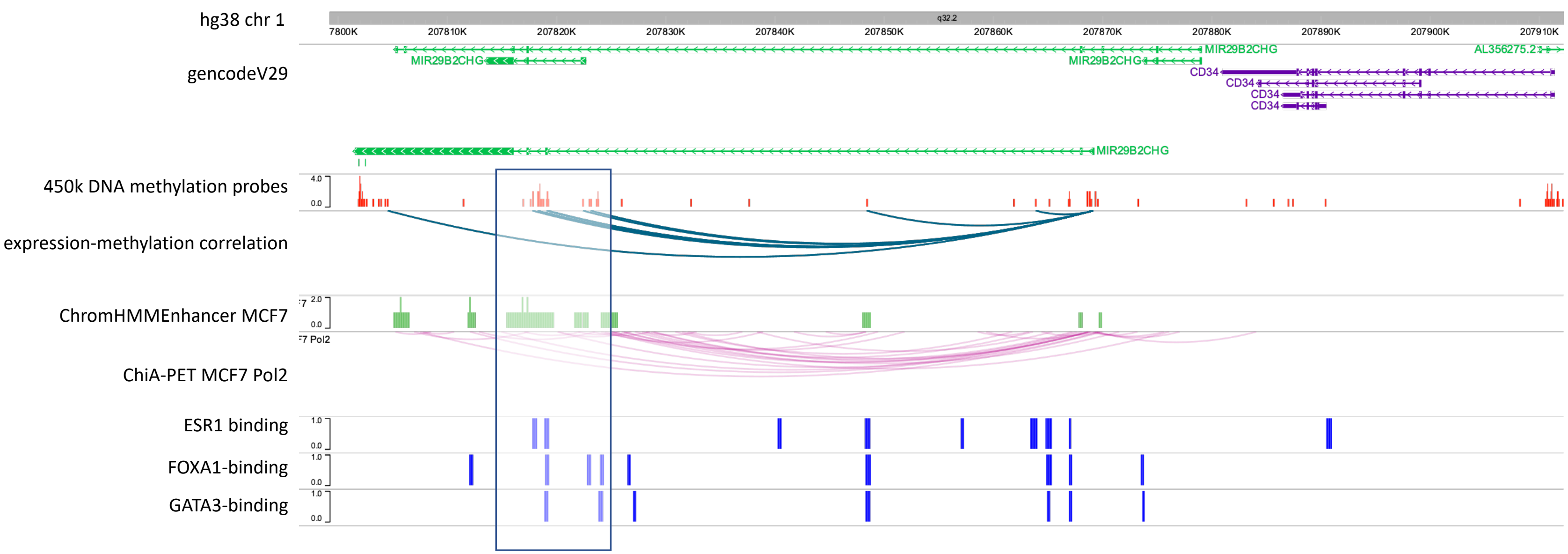

Supplementary Figure 15. Distal regulatory element in lncRNA loci

Graphical illustration of the *MIR29BCHG* locus annotated for different epigenomic tracks. CpGs measured by the 450k Illumina array are shown together with the significant negative correlations between levels of DNA methylation and lncRNA expression in the OSL2 and TCGA cohorts (blue arcs, expression-methylation correlation). ChromHMM Enhancer regions (active and genic) in the Mcf7 cell line (green), ChiA-PET polII loop connecting the TSS of the respective lncRNA to the CpG in the enhancer region. TF binding of ESR1 (dark blue), GATA3 (light blue), and FOXA1 (grey blue) from ChIP-seq experiments (ReMap).
