# Supplementary material for: Subtype and cell type specific expression of lncRNAs provide insight into breast cancer
Source: Commun Biol. 2022 Aug 18;5:834. doi: 10.1038/s42003-022-03559-7 (PMC9388662; doi:10.1038/s42003-022-03559-7)
Supplement: Supplementary file 13 — Reporting Summary [file 42003_2022_3559_MOESM13_ESM.pdf]

nature portfolio

Corresponding author(s): Vessela Kristensen and Xavier Tekpli

Last updated by author(s): May 6, 2022

Reporting Summary

Nature Portfolio wishes to improve the reproducibility of the work that we publish. This form provides structure for consistency and transparency in reporting. For further information on Nature Portfolio policies, see our Editorial Policies and the Editorial Policy Checklist.

Statistics

For all statistical analyses, confirm that the following items are present in the figure legend, table legend, main text, or Methods section.

n/a	Confirmed	
☐	☒	The exact sample size (*n*) for each experimental group/condition, given as a discrete number and unit of measurement
☐	☒	A statement on whether measurements were taken from distinct samples or whether the same sample was measured repeatedly
☐	☒	The statistical test(s) used AND whether they are one- or two-sided<br>*Only common tests should be described solely by name; describe more complex techniques in the Methods section.*
☐	☒	A description of all covariates tested
☐	☒	A description of any assumptions or corrections, such as tests of normality and adjustment for multiple comparisons
☐	☒	A full description of the statistical parameters including central tendency (e.g. means) or other basic estimates (e.g. regression coefficient) AND variation (e.g. standard deviation) or associated estimates of uncertainty (e.g. confidence intervals)
☐	☒	For null hypothesis testing, the test statistic (e.g. *F*, *t*, *r*) with confidence intervals, effect sizes, degrees of freedom and *P* value noted<br>*Give P values as exact values whenever suitable.*
☒	☐	For Bayesian analysis, information on the choice of priors and Markov chain Monte Carlo settings
☒	☐	For hierarchical and complex designs, identification of the appropriate level for tests and full reporting of outcomes
☐	☒	Estimates of effect sizes (e.g. Cohen's *d*, Pearson's *r*), indicating how they were calculated

Our web collection on statistics for biologists contains articles on many of the points above.

Software and code

Policy information about availability of computer code

Data collection	Quantification of gene expression was performed using kallisto (v0.46.0).
Data analysis	All analysis was performed in the R software environment (v4) unless otherwise specified. Specific R packages used for downstream analysis: tximport v1.10.1 and v1.16.1, pheatmap (v1.0.12), TCGAbiolinks (V2.16.3), edgeR (v3.24.3 (TCGA)/3.30.3 (SCAN-B)), Limma/voom R-package (v 3.38.3/3.44.3), Survival (v3.3-1), Recount3 (v 1.2.6), Seurat (v3.2.1 ans v4.1.0), BioMart (biomaRt_2.45.6), LOLA (v1.12.0). Other software: blast (ncbi-blast-2.6.0), Nanodissect (http://nano.princeton.edu/), BEDtools (v2.29.2), UniBind enrichment tool (https://unibind.uio.no/enrichment/, source code available at https://bitbucket.org/CBGR/unibind_enrichment/; input R data with TFBS information available on zenodo at https://doi.org/10.5281/zenodo.4452896), UCSC liftOver (https://genome.ucsc.edu/cgi-bin/hgLiftOver)

For manuscripts utilizing custom algorithms or software that are central to the research but not yet described in published literature, software must be made available to editors and reviewers. We strongly encourage code deposition in a community repository (e.g. GitHub). See the Nature Portfolio guidelines for submitting code & software for further information.

Data

Policy information about availability of data

All manuscripts must include a data availability statement. This statement should provide the following information, where applicable:

- Accession codes, unique identifiers, or web links for publicly available datasets
- A description of any restrictions on data availability
- For clinical datasets or third party data, please ensure that the statement adheres to our policy

Provide your data availability statement here.

Field-specific reporting

Please select the one below that is the best fit for your research. If you are not sure, read the appropriate sections before making your selection.

☒ Life sciences ☐ Behavioural & social sciences ☐ Ecological, evolutionary & environmental sciences

For a reference copy of the document with all sections, see nature.com/documents/nr-reporting-summary-flat.pdf

Life sciences study design

All studies must disclose on these points even when the disclosure is negative.

Sample size	SCAN-B(n=3455), TCGA(n=1095), Oslo2(n=279). Cohort study including all patients with relevant molecular data for the described analysis.
Data exclusions	Duplicate samples were removed, otherwise no data was excluded from the analysis
Replication	Results emphasize findings that were concordant in two independent cohorts.
Randomization	NA
Blinding	NA

Reporting for specific materials, systems and methods

We require information from authors about some types of materials, experimental systems and methods used in many studies. Here, indicate whether each material, system or method listed is relevant to your study. If you are not sure if a list item applies to your research, read the appropriate section before selecting a response.

Materials & experimental systems

n/a	Involved in the study	
☒	☐	Antibodies
☒	☐	Eukaryotic cell lines
☒	☐	Palaeontology and archaeology
☒	☐	Animals and other organisms
☐	☒	Human research participants
☒	☐	Clinical data
☒	☐	Dual use research of concern

Methods

n/a	Involved in the study	
☒	☐	ChIP-seq
☒	☐	Flow cytometry
☒	☐	MRI-based neuroimaging

Human research participants

Policy information about studies involving human research participants

Population characteristics	The SCAN-B cohort has been described in detail in Vallon-Christersson et. al. 2019 https://doi.org/10.1038/s41598-019-48570-x<br>The Olso2 cohort has been described in detail in Aure et. al 2017 https://doi.org/10.1186/s13058-017-0812-y
Recruitment	Patients with primary invasive disease were enrolled in SCAN-B between September 1 2010 and March 31 2015 , fresh tumor samples taken by a pathologist during the clinical diagnostic routine.<br>The Oslo2 breast cancer cohort is a consecutive study collecting material from breast cancer patients with primary operable disease at several hospitals in south-eastern Norway.
Ethics oversight	SCAN-B: Regional Ethical Review Board in Lund, Sweden (Registration numbers 2009/658, 2010/383, 2012/58, and 2013/459)<br>Oslo2: Norwegian Regional Committee for Medical Research Ethics (approval number 1.2006.1607, amendment 1.2007.1125)

Note that full information on the approval of the study protocol must also be provided in the manuscript.
